# Neuropharmacological Potential and Delivery Prospects of Thymoquinone for Neurological Disorders

**DOI:** 10.1155/2018/1209801

**Published:** 2018-03-20

**Authors:** Md. Jakaria, Duk-Yeon Cho, Md. Ezazul Haque, Govindarajan Karthivashan, In-Su Kim, Palanivel Ganesan, Dong-Kug Choi

**Affiliations:** ^1^Department of Applied Life Science, Graduate School, Konkuk University, Chungju 27478, Republic of Korea; ^2^Research Institute of Inflammatory Disease, Department of Biotechnology, College of Biomedical and Health Science, Konkuk University, Chungju 27478, Republic of Korea; ^3^Nanotechnology Research Center, Konkuk University, Chungju 27478, Republic of Korea

## Abstract

Thymoquinone (TQ) is an active ingredient isolated from *Nigella sativa* and has various pharmacological activities, such as protection against oxidative stress, inflammation, and infections. In addition, it might be a potential neuropharmacological agent because it exhibits versatile potential for attenuating neurological impairments. It features greater beneficial effects in toxin-induced neuroinflammation and neurotoxicity. In various models of neurological disorders, it demonstrates emergent functions, including safeguarding various neurodegenerative diseases and other neurological diseases, such as stroke, schizophrenia, and epilepsy. TQ also has potential effects in trauma mediating and chemical-, radiation-, and drug-induced central nervous system injuries. Considering the pharmacokinetic limitations, research has concentrated on different TQ novel formulations and delivery systems. Here, we visualize the neuropharmacological potential, challenges, and delivery prospects of TQ, specifically focusing on neurological disorders along with its chemistry, pharmacokinetics, and toxicity.

## 1. Introduction

Thymoquinone (TQ) is a major bioactive compound mainly found in the *Nigella sativa* L. [1–3]. *N. sativa* is an emerging miracle herb that belongs to the Ranunculaceae family and is known as an annual herbaceous plant that is frequently grown in Western Asia, Middle East, Eastern Europe, and Mediterranean countries [4, 5]. Traditionally, the seeds of *N. sativa* are used in the Middle East, India, and Northern Africa for the treatment of cough, asthma, bronchitis, influenza, fever, headache and rheumatism [6–8]. Furthermore, it is also employed as an appetite stimulant, digestive, liver tonic, antidiarrheal, and emmenagogue to increase milk production in a lactating mother. Also, it has an immune system protecting activity against parasitic infections [5].

Growing evidence concerning the versatile TQ pharmacological activities has been established. In a number of preclinical studies, it elicits antitussive, gastroprotective, anti-inflammatory, antinociceptive, antihistaminic, antibacterial, anthelmintic, antioxidant, immunomodulatory, anticancer, hepatoprotective, cardioprotective, antidiabetic, ototoxicity protective, and nephroprotective effects [9–31]. *N. sativa* and TQ have numerous advantageous properties with relevance to various neurological illnesses. For instance, they have anticonvulsant, anxiolytic, antidepressant, and antipsychotic potential. They also counter memory impairments and enhance cognitive functioning as well as attenuate drug tolerance and dependence [32–47]. Moreover, many recent studies have described the neuropharmacological properties of TQ such as anti-inflammatory and antioxidative roles in designed neurological models [48–50]. Besides, it alleviates neuropathy in an experimental peripheral diabetic model [51]. Yet, the pharmacological potential and delivery prospects of TQ in neurological illnesses have not been documented to date. Therefore, in this article, considering the neurological disorders, we deliberate the neuropharmacological potential, challenges, and delivery concepts of TQ along with its chemistry, pharmacokinetics, and toxicity.

## 2. Chemistry of Thymoquinone

**Figure 1:**
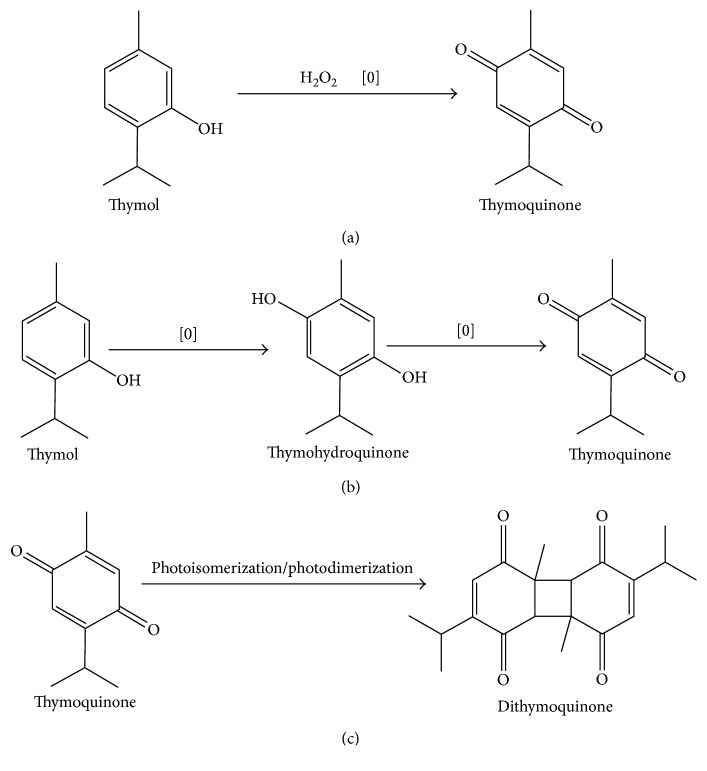
(a) Oxidative conversion of thymoquinone from thymol. (b) Hypothesized mechanism of transition between quinones by oxidation under controlled heat. (c) Conversion of dithymoquinone from thymoquinone by photoisomerization or photodimerization reactions.

Thymoquinone is a monoterpene diketone and an important bioactive compound that forms 18.4%–24% of these essential oils with a boiling point and melting point of 230–232°C and 44‐45°C, respectively. Its molecular weight is 164.204 g/mol, and value of Log *P* is 2.20 denoting lipophilicity of TQ. Moreover, it has the ability to penetrate the blood-brain barrier (BBB) owing to its molecular weight (less than 500 g/mol) and Log *P* (less than five) value. Thus, it might be suitable for clinical trials [52–56]. Structurally, it is homologous with coenzyme Q, an important antioxidant of the electron transport chain [57]. By oxidation of thymol with hydrogen peroxide, TQ can be readily synthesized in gram quantities [58]. Moreover, according to one hypothesis based on chemical composition, oxidation of quinone compounds caused by thermal processing leads to modification and conversion between compounds and accumulation of more powerful compounds. The controlled heating causes oxidation of thymol and converts it into thymohydroquinone. The heating process persistence leads to a further oxidative process that converts thymohydroquinone into thymoquinone, resulting in the accumulation of larger amounts of TQ. Based on the presence of light, the photoisomerization of TQ may cause an accumulation of its dimer, dithymoquinone [57]. Besides this, during quinone separation and extraction from seeds, thymoquinone photodimerization as a consequence of exposure to sunlight produces dithymoquinone [11]. Conversions of different quinones by the chemical reaction are shown in Figure 1.

## 3. Pharmacokinetics and Toxicity of Thymoquinone

Thymoquinone's pharmacokinetic profiles have been reported in several studies [59, 60]. Its solubility in water has reported as <1.0 mg/ml at room temperature [61]. TQ has poor oral bioavailability based on its low aqueous solubility and dissolution rate [62]. Moreover, TQ represents a compound with rapid plasma concentration-time curves demonstrating a rapid polyexponential decline following intravenous dosing [63]. The plasma concentration of glibenclamide increases through the coadministration of TQ as single and multiple doses, respectively [64]. In addition, TQ binds with major plasma proteins including bovine serum albumin (BSA) and alpha-1 acid glycoprotein (AGP) [65–68]. Molecular modeling and fluorescence quenching studies have confirmed the binding interaction of TQ with BSA and AGP [65, 66]. Through covalent bonding, TQ binds to both BSA and AGP. Covalent binding of TQ to BSA leads to eliminating the TQ anticancer activity against various tested cancer cell lines, while on the other hand, TQ anticancer activity was not affected when TQ is bound to AGP [67, 68]. There could be biotransformation owing to the metabolizing activity of liver enzymes, such as DT-diaphorase (a quinine reductase), which catalyzes the reduction of TQ into a dihydrothymoquinone [69]. This metabolite exhibits antioxidant properties that are more potent than those of TQ and similar to those of Trolox, which considers a standard antioxidant compound [70]. With this, TQ-treated diabetic rats show a marked decrease in hepatic protein expressions of cytochrome P450 3A2 and cytochrome P450 2C11 enzymes that are responsible for the metabolism of glibenclamide. At the glucose level, TQ has a synergistic effect with glibenclamide, which could be explained by reduction cytochrome P450 activity at the protein level. In addition, the area under the plasma drug concentration-time curve and plasma half-life of glibenclamide were also elevated, respectively, by a single dose of TQ as well as after chronic treatment [64].

A variety of *in vitro* and *in vivo* studies has evaluated thymoquinone toxicity profile [71, 72]. The oral and intraperitoneal (i.p.) median lethal doses (LD_50_) for TQ in rats as well as in mice were successfully determined by several research groups [73, 74]. At higher doses, it is well tolerated by male and female rats. TQ at 10 mg/kg body weight was found to have no observed adverse effect level [75]. Moreover, the administration of 20 ml develops a TQ-rich fraction nanoemulsion per kg that was not toxic following acute exposure in Sprague-Dawley rats [76]. In terms of acute toxicity, TQ-loaded nanostructured lipid carriers (TQ-NLC) have less toxicity than pure TQ. In a subacute toxicity study, oral administration of 100 mg/kg of TQ-NLC caused mortality in neither male nor female mice but resulted in liver toxicity. For long-term oral consumption, TQ and TQ-NLC at doses of 10 mg/kg are safe in mice. These results provided safety information pertaining to TQ-NLC, which further aids researchers in clinical use [77]. Furthermore, another study determined LD_50_ = 104.7 mg/kg in mice i.p. and 870.9 mg/kg after oral ingestion of TQ. In rats, the value of LD_50_ was detected to be 57.5 mg/kg while it was 794.3 mg/kg after i.p. and oral administration, respectively [73]. Interestingly, using TQ in combination with ionizing radiation such as *γ*-radiation was found to exert a synergistic cytotoxic effect on breast cancer cells *in vitro* [78]. Cisplatin administration and a high dose of TQ act synergistically to produce nephrotoxicity where the involvement of the apoptotic pathway and proximal tubule damage might be a primary determinant of this effect [79].

## 4. Neuropharmacological Potential of Thymoquinone

### 4.1. Activities in Neuroinflammation Model

Neuroinflammation is the term used to describe the inflammatory response originating in the central nervous system (CNS) after suffering an injury, where there is an accumulation of glial cells (microglia and astrocytes) [80]. TQ possesses antineuroinflammatory activity, specifically via lipopolysaccharide- (LPS-) induced BV-2 microglial cells [49, 81, 82]. It is effective in reducing nitrite (NO^2−^) with a half maximal inhibitory concentration (IC_50_) of 5.04 *μ*M, relative to selective inducible nitric oxide synthase (iNOS) inhibitor LNIL-L-N6-(1-iminoethyl) lysine (IC_50_ = 4.09 *μ*M). TQ reduces the level of NO^2−^ found to parallel the decline of iNOS protein expression. In activated microglia, TQ (10 *μ*M) attenuates the increased levels of proinflammatory cytokines/chemokines including interleukin- (IL-) 6, IL-12p40/70, chemokine (C-C motif) ligand 12/monocyte chemotactic protein 5, chemokine (C-C motif) ligand 2/monocyte chemoattractant protein 1, and granulocyte colony-stimulating factor. TQ has also been observed to mediate attenuation of monocyte chemotactic protein 5, monocyte chemoattractant protein 1, and IL-6 protein in supernatants from activated BV-2 cells. In addition, TQ reduces LPS-mediated elevation in gene expression of C-X-C motif chemokine 10 and a number of other cytokines in the panel [81]. Considering the recent study on nuclear factor E2-related factor 2 (Nrf2)/antioxidant responsive element signaling pathway, TQ activates this signal by elevating the nuclear localization, DNA binding, and Nrf2 transcriptional activity, along with increasing protein levels of heme oxygenase-1 (HO-1) and NAD(P)H:quinone oxidoreductase. In this study, it also inhibits nuclear factor kappa-light-chain-enhancer of activated B cell- (NF-*κ*B-) dependent neuroinflammation in BV-2 microglia [82].

Regarding recent mechanistic studies, TQ produces antineuroinflammatory activity through the AMP-activated protein kinase (AMPK) and nicotinamide adenine dinucleotide (oxidized)/sirtuin 1 (SIRT1) pathways. Further, TQ diminishes cellular ROS generation, possibly through inhibition of p40^phox^ and gp91^phox^ protein. Treatment of BV-2 microglia with TQ also results in an elevation in the levels of liver kinase B1 and phospho-AMPK proteins. Furthermore, TQ reduces cytoplasmic levels and increases nuclear accumulation of SIRT1 protein and enhances nicotinamide adenine dinucleotide (oxidized) concentration. Besides this, the effect of RNAi or pharmacological antagonists suppressing the anti-inflammatory activity of TQ was abolished with expressions of AMPK and SIRT1. Pharmacological antagonism of AMPK reverse TQ-induced increases in SIRT1 [49]. Another recent investigation has offered evidence for TQ's anti-inflammatory activity. At different concentrations, TQ strongly inhibits the nitric oxide (NO) production and represses iNOS, tumor necrosis factor- (TNF-) *α*, cyclooxygenase-2, IL-6, and IL-1*β* expression in LPS-activated RAW264.7 cells [83]. Moreover, the protective effects of TQ on LPS-induced brain tissues oxidative stress status induced in rats were reported. Compared with a control group, LPS elevates malondialdehyde (MDA) and NO metabolites while decreasing thiol content, superoxide dismutase (SOD), and catalase (CAT) in both the hippocampus and cortex. Treatments with TQ (2, 5, or 10 mg/kg) decrease IL-6, TNF-*α*, MDA, and NO metabolites and increase thiol content along with SOD and CAT compared to the LPS group [84].

### 4.2. Activities in Induced Disease Models

#### 4.2.1. Model of Alzheimer's Disease

Alzheimer's disease (AD) is a chronic neurodegenerative disorder where the major risk factor is age with data showing a considerable increase in incidence after the age of 60 years [85, 86]. Its impact on public health and society as a whole is devastating [86]. It is also known as the most common type of age-related dementia and is characterized by memory loss, cognitive decline, and debilitating neurodegeneration [86–88].

Regarding research studies, TQ might be a potential agent against AD. In a model of amyloid beta- (A*β*-) induced neurotoxicity, cultured hippocampal and cortical neurons were treated with A*β*_1–42_ and TQ simultaneously for 72 hours. Treatment with TQ (100 nM) efficiently attenuates A*β*_1–42_-induced neurotoxicity, as evidenced by improving cell viability. A*β*_1–42_ causes mitochondrial membrane potential depolarization and reactive oxygen species (ROS) generation while TQ inhibited these changes. In addition, TQ restores synaptic vesicle recycling inhibition, partially upturns the loss of spontaneous firing activity, and also inhibits A*β*_1–42_ aggregation *in vitro* [89]. Another study described the inhibitory effects of TQ in comparison to tannic acid (TA) on A*β* fibril formation. The effects of TQ on the formation of A*β*_1–40_ were studied spectrophotometerically, over a duration of 16 days at a pH of 7.2 and 37°C. TQ and TA, with an IC_50_ of 0.1 and 29 *μ*M at day 0 (within 5 h), respectively, inhibit A*β* aggregation in a dose-dependent way at different concentrations (1, 10, and 50 *μ*M). However, at the day 16, IC_50_ of TQ and TA was found to be 0.2 and 0.01 *μ*M, respectively. In an electron microscopic study, coincubation of TQ with A*β* reduces the numbers of fibrils to a certain degree with shorter fibrils and small amorphous aggregates. TQ pretreatment protects A*β*_1–40_ cytotoxic effects in primary cultured cerebellar granule neurons [90]. Furthermore, in combination with *α*7 nicotinic acetylcholine receptor's positive allosteric modulators (PAM) with a known agonist (PNU-282987) or with TQ as a possible treatment for AD in a rat model has evaluated. LPS causes acidophilic masses, deformed neurons, Congo red (+ve) masses, and reduced cAMP response element-binding protein (CREB) phosphorylation (phospho-CREB) immunoexpression. Concerning histological, histochemical, and immunohistochemical studies, TQ or *α*7 nicotinic acetylcholine receptor agonist combined with PAM can have an important role in treatment of AD [91]. In a recent study, TQ improves LPS-induced learning and memory impairments in rats by attenuating hippocampal cytokine levels and brain tissue oxidative damage [84].

#### 4.2.2. Model of Parkinson's Disease

Parkinson's disease (PD) is considered a progressive neurodegenerative disorder characterized by the dopaminergic neuron loss in the substantia nigra pars compacta (SNpc) and neuroinflammation through microglial malfunction [92, 93]. TQ exhibits protective activities in PD models. The neuroprotective effects of TQ on primary dopaminergic cultures from mouse mesencephalon using 1-methyl-4-phenylpyridinium (MPP^+^) and rotenone toxicities were studied previously [94, 95]. MPP^+^ (10 *μ*M on day 10 *in vitro* for 48 h) significantly decreases the number of tyrosine hydroxylase immunoreactive (THir) by 40% compared with untreated control cultures. Rotenone with both short- (20 nM on day 10 *in vitro* for 48 h) and long-term (1 nM on day six *in vitro* for six consecutive days) toxicities reduces the number of THir neurons by 33% and 24%, respectively. Treatment of cultures with TQ (0.01, 0.1, 1, and 10 *μ*M on day eight for four days) rescues approximately 25% of THir neurons at concentrations of 0.1 *μ*M and 1 *μ*M against MPP^+^-induced cell death. Against rotenone, TQ afforded significant protection in both short- and long-term models. In case of short-term rotenone toxicity, TQ (from days 8 to 12) saves about 65%, 74%, and 79% of THir neurons at concentrations of 0.01, 0.1, and 1 *μ*M, respectively, compared with cell loss induced by rotenone. In long-term rotenone toxicity scenarios, concomitant treatment of cultures with TQ significantly rescues about 83 to 100% of THir neurons compared with rotenone-treated cultures [94]. Additionally, MPP^+^ decreases the number of dopaminergic neurons by 40% and enhances the release of lactate dehydrogenase (LDH) into the culture medium. TQ significantly rescues dopaminergic neurons and decreases the release of LDH at the concentrations of 0.1 and 1 *μ*M. Moreover, TQ treatment significantly shifts the red fluorescent intensity of the LysoTracker® Deep Red, increasing the mitochondrial membrane potential as it increased the red: green fluorescent ratio of JC-1 and decreases MPP^+^-induced apoptotic cell death. TQ is thought to protect dopaminergic neurons in primary mesencephalic cultures by enhancing lysosomal degradation that clears damaged mitochondria and inhibits mitochondria-mediated apoptotic cell death [95].

Furthermore, TQ produces neuroprotective activity in a 6-hydroxydopamine- (6-OHDA-) induced PD model. Unilateral intrastriatal 6-OHDA-lesioned rats were daily pretreated per oral (p.o.) with TQ at doses of 5 and/or 10 mg/kg three times at an interval of 24 h. TQ pretreatment significantly improves turning behavior, prevents loss of SNpc neurons, and lowers level of MDA. Hence, TQ offers neuroprotection against 6-OHDA-induced neurotoxicity that is partly based on the attenuation of lipid peroxidation [96]. Regarding the recent study, TQ (7.5 and 15 mg/kg/day, p.o.) pretreatment was administered one hour prior to rotenone injection, affecting motor test results (rotarod, rearing, and bar tests). TQ significantly prevents rotenone-induced motor defects and modifications in the Parkin, dynamin-related protein 1, dopamine, and tyrosine hydroxylase levels in the studied areas [97]. A summary of the effects of TQ in PD models is located in Table 1.

#### 4.2.3. Model of Autoimmune Encephalomyelitis

Experimental autoimmune encephalomyelitis (EAE) is a CD4^+^ T cell-mediated inflammatory demyelinating condition of the CNS. It is frequently employed as a model of multiple sclerosis, because this model has great importance for understanding multiple sclerosis pathogenesis and its therapy design [98, 99]. In the EAE experimental model, oxidative stress plays a significant role in EAE onset and progression. Therefore, reducing oxidative stress might ameliorate EAE sign and symptoms. Through utilizing myelin basic protein emulsified with complete Freund's adjuvant, EAE was induced in female Lewis rats and TQ (1 mg/kg) was injected into the tail vein. Oxidative stress inhibition is observed, and TQ might improve the symptoms in EAE animals [100]. TQ concomitant with myelin basic protein after the appearance of clinical signs resulted in preventing and ameliorating EAE. TQ (1 mg/kg per day) was able to counter perivascular cuffing and infiltration of mononuclear cells in the brain and spinal cord, increase the red blood cell glutathione (GSH), and inhibit the activation of NF-*κ*B in the brain and spinal cord [101]. In addition, in a myelin oligodendrocyte glycoprotein- induced EAE model, the antioxidant effect of TQ has nearly 90% preventive and 50% therapeutic effects in chronic relapsing EAE [102].

#### 4.2.4. Model of Stroke

Ischemia/reperfusion (I/R) injury is the cascade of events leading to neuronal injury and death. It involves NO, excitatory amino acids, cytokine and free radical release, mitochondrial respiratory enzyme damage, induction of programmed cell death, and activation of microglia [103]. It is known that redox imbalance, inflammation, and apoptosis are the major mechanisms of I/R injury [104]. TQ shows potential activity against I/R injury and neuronal damage. Five days before ischemia, TQ (5 mg/kg) administered orally and sustained during reperfusion attenuated forebrain ischemia-induced neuronal damage. TQ treatment significantly diminishes the dead hippocampal neuronal cell number (24% in TQ-treated versus 77% for ischemia), thereby supporting TQ's protective role in I/R injury. Accordingly, TQ also shows an effect against oxidative stress [105]. Moreover, treatment with TQ (10 mg/kg i.p.) for seven days before induction of spinal cord I/R injury improves neurological outcomes. Specifically, TQ produces effect against oxidative stress and neuroinflammation and also reduces the motor neuron apoptosis. TQ treatment also shows preservation of tissue structure [50]. With this, after TQ administration via the noninvasive nasal route of TQ mucoadhesive nanoemulsion improves neurobehavioral activity (locomotor and grip strength) was observed in middle cerebral artery occlusion-induced cerebral ischemia [106]. Additionally, in a rodent cerebral I/R model, TQ-loaded PLGA-chitosan nanoparticles optimized delivery through the nose-to-brain route exhibit the neuroprotective efficacy against cerebral ischemia [107].

#### 4.2.5. Schizophrenia Model

The mental disorder, schizophrenia, is characterized by severe cognitive impairment, as well as positive and negative symptoms such as hallucination, delusion, thought disorders, apathy, and social withdrawal [108, 109]. Moreover, regarding the adverse effects of available antipsychotic medications, recent investigations have focused on the search for well-tolerated, safe molecules from natural sources to control the severity and progression of the disorder [110]. A potential role of TQ in combating schizophrenia has been described. With this, TQ demonstrates potential activity against schizophrenia in mouse models. In one study for 28 days, i.p. doses of TQ (20 mg/kg) were administered. The pretreatment of TQ alone and in combination with haloperidol led to observed cataleptic behavior. Moreover, in an apomorphine-induced climbing behavior model, the administration of TQ significantly reduces the ultimate time taken for single climb and climbing index. TQ reduces scopolamine-induced prolongation of transfer latency. However, a significant rise in possible alternation was observed, suggesting its antiamnesic effects. TQ possesses antiamnesic effect with a decrease in acetylcholinesterase (AChE) enzymatic activity in mouse brain. Decreased concentration of thiobarbituric acid reactive substance (TBARS) and the rise in glutathione and CAT levels were observed in all models [40]. Additionally, TQ administration also reduces dopamine levels, indicating the involvement of dopamine receptors in all three models, hence, demonstrating its antipsychotic-like activities [40].

#### 4.2.6. Model of Epilepsy

Epilepsy is a brain disorder characterized by convulsive seizures or loss of consciousness or both and induced by a complex of neurotransmitter systems [111, 112]. There is growing interest in the use of natural sources to find treatments.

A number of investigations have reported the antiepileptic activity of TQ. Pentylenetetrazole- (PTZ-) induced epilepsy is a common model for investigating epilepsy, and in this model, TQ yields antiepileptic activity [113–116]. With PTZ-induced status epilepticus (SE), intracerebroventricular (i.c.v.) administration of TQ (200 and 400 *μ*M) extended the time until onset and decreased tonic-clonic seizure duration. TQ protection against lethality was 45% and 50% in the respective doses. In this study, flumazenil (1 nM i.c.v.) reverses the anticonvulsant activity of TQ. Also, pretreatment with naloxone (10 *μ*M i.c.v.) antagonizes the tonic-clonic seizure latency prolongation and the reduction in duration of seizure induced by TQ at dose of 200 *μ*M. The anticonvulsant activity of TQ is probably through an opioid receptor-mediated increase in GABAergic tone [113]. Moreover, another study reported the antiepileptic potential of TQ in mice using PTZ and maximal electroshock seizure- (MES-) induced convulsion models. TQ in both PTZ and MES models increased sodium valproate potency (SVP). In addition, TQ reduces the ED_50_ of the SVP in both the models. However, at both 50 and 100 mg/kg doses, TQ significantly potentiates SVP antiepileptic response in PTZ model [114]. Furthermore, TQ along with phenobarbital combination therapy produces additive anticonvulsant effect compared to monotherapy with phenobarbital in PTZ-induced rat model [115]. According to the another study, TQ and vitamin C exhibit anticonvulsant effects via activation of the gamma-aminobutyric acid B1 receptor (GABAB1R)/calcium/calmodulin-dependent protein kinase II (CaMKII)/CREB pathway, suggesting a potential therapeutic role in epilepsy. Animals pretreated with either p.o. administration of TQ (40 mg/kg administered for seven days) or vitamin C (vitamin C 250 mg/kg i.p. 2 h before PTZ i.p. injection) or in combination (vitamin C 250 mg/kg i.p. and TQ 40 mg/kg orally 2 h before PTZ). Compared to PTZ, TQ and vitamin C significantly prolong the onset of seizures, as well decrease the incidence of high-grade seizures. Electroencephalogram (EEG) analysis indicates that TQ or vitamin C supplementation significantly reduces polyspike and epileptiform discharges. In comparison with the control group, SE cause a decline in GABAB1R expression but no change expression of protein kinase A. Additionally, seizures also decrease CaMKII and prevent phospho-CREB in both the cortex and hippocampus areas, respectively. Meanwhile, TQ and vitamin C supplementation reversed the PTZ-induced changes in expression of GABAB1R, CaMKII, and CREB. Moreover, PTZ significantly increases Bcl-2-associated X protein (Bax) concentrations, decreases a crucial antiapoptotic protein and B-cell lymphoma protein-2 (Bcl-2) expression, and diminishes the activation of caspase-3 [116]. In a penicillin-induced epilepsy model, TQ also has antiepileptic activity in rats. Various doses of TQ (10, 50, and 100 mg/kg) significantly increase the latency time to the onset of the first spike wave and decrease the frequency and amplitude of epileptiform activity over the first 20 minutes compared with the control group [33]. In another epileptic model, kainic acid (0.5 *μ*g/ventricle i.c.v.) bilaterally induces SE in male Wistar rats. The lesioned rats were treated with TQ (10 mg/kg i.p.) for four days. TQ significantly decreases suppression of spontaneous recurrent motor seizures, reduces neuron degeneration (25%) in CA1, CA3, and the dentate hilus, and suppresses mossy fiber sprouting (30–40%) both in four-month and 12-month age groups. TQ also increases neurogenesis in both groups; however, in middle-aged rats, it is significantly more than in young rats [117].

Recently, in a lithium-pilocarpine rat model, the protective impact of TQ is observed in brain injury of SE. TQ pretreatment (10 mg/kg twice) latency to SE increases compared with rats in the model group, whereas the total power was suggestively lesser. According to the behavioral experiments, TQ might also have a protective outcome on the function of learning and memory. Additionally, pretreatment with TQ significantly improves the Nrf2 and HO-1 protein expression as well as SOD in the hippocampus [48]. TQ activities in epilepsy models are described in Table 2.

#### 4.2.7. Models of Depression and Anxiety

Regarding the reported studies, TQ possesses both antidepressant and anxiolytic activities according to mouse models. In one mouse model of depression, TQ modulates the levels of neurotransmitters and reduces oxidative stress. GSH level reduces and TBARS level increases in a modified forced swim test and tail suspension test in mice. Pretreatment of TQ (20 mg/kg) restores GSH and decreases TBARS levels. TQ combination with fluoxetine (10 mg/kg) also led to a reduction in TBARS and elevates GSH levels [39]. Moreover, TQ yields protective effects against oxidative stress via restraint stress-induced biochemical alterations in Wistar albino rats [118]. Both the doses of TQ (5 and 10 mg/kg) treatment significantly ameliorate restraint stress-induced increased IL level and enzymatic activities in serum. In the brain, restraint stress significantly increases TBARS, NO^2−^, and nitrate levels and TQ treatment brought back the concentration back to the normal levels. Besides, restraint stress significantly decreases oxidative enzyme activities, such as those of the SOD, CAT, glutathione-s-transferase, glutathione peroxidase (GSH-Px), and glutathione reductase (GR). With this, TQ treatments significantly enhance the activities compared to untreated stressed rats [118]. Furthermore, in the unstressed mice, TQ (10 and 20 mg/kg) elicits the anxiolytic effects without changing NO^2−^ levels, but only at the higher dose (20 mg/kg) improving the GABA content. On the other hand, in the stressed mice, TQ (20 mg/kg) produces anxiolytic effects with a major reduction in plasma NO^2−^ and reversal of the reduced brain GABA content. Nitric oxide-cyclic guanosine monophosphate and GABAergic pathways are considered crucial in the anxiolytic activities of TQ [36].

#### 4.2.8. Model of Neuropathic Pain

At present, pain is one of the main health problems among the world's population. It is also one of the most pervasive issues of high social and economic impacts in society [119–121]. The generation of neuropathic pain is through lesions to the somatosensory nervous system that changes its structure and function so that pain happens spontaneously and via responses to noxious and innocuous stimuli that pathologically amplified [122]. TQ has potential activity against neuropathic pain. Moreover, the role of oxidative stress, spinal glial activation, and cell death is involved in the pathogenesis of neuropathic pain. Repeated treatment with TQ (2.5 and 5 mg/kg) significantly alleviates behavioral signs of neuropathic pain. In the lumbar spinal cord of neuropathic rats, repeated TQ administration reduces elevated Bax and ionized calcium-binding adapter molecule in terms of concentrations on day three, while stimulating the production of Bcl-2. In addition, ionized calcium-binding adapter molecule and Bax/Bcl-2 ratio have declined by days 7 and 14 as a consequence. Furthermore, TQ treatment (2.5 and 5 mg/kg) restores the levels of MDA, and a high dose of TQ (5 mg/kg) also reverses the decreased GSH in injured animals. Microglia, apoptotic factors, and oxidative stress contribute to the pathogenesis of chronic constrictive injury, and TQ plays an antinociceptive role, possibly through antioxidant and antineuroinflammatory effects [123]. In another study, TQ shows potent activity against neuropathic pain in a spinal cord injury model. Various doses of TQ, such as 100 mg/kg, 200 mg/kg, and 400 mg/kg, were used. At the beginning of the experiment, spinal cord injury was applied to all groups except the sham group following a mechanical and heat-cold test. From the mechanical and heat-cold allodynia measurements, the withdrawal threshold and withdrawal latency values were recorded for all three groups receiving TQ and these values were higher than those of the control group at all time points. All of the TQ treatment groups increased the paraoxonase and total antioxidant concentrations, while there was a decrease in NO, MDA, IL-1*β*, TNF-*α*, and total oxidants levels [124]. Additionally, TQ antinociceptive activity and among its structural analogs have been described. Previously, the quinones were prepared by an oxidation procedure using molecular oxygen and catalysis with [CoII(salen)] from the respective phenols. The antinociceptive activity of *para*-benzoquinones (10 mg/kg i.p.) was evaluated using a formalin test in mice. Regarding the tested compounds, five *para*-benzoquinones exhibited antinociceptive activity. The 2-isopropyl-*para*-benzoquinone presented the highest potency in the first and second phases and produced near-maximal inhibition during the formalin test, similar to morphine. The appropriate structural modification of *para*-benzoquinones may make it possible to develop novel analgesic drugs [125].

## 5. Miscellaneous Activities

### 5.1. Against Traumatic Brain and Spinal Cord Injuries

As a highly complex disorder, traumatic brain injury is the leading cause of mortality and disability in people under the age of 45 years [126, 127]. Spinal cord injury, on the other hand, is quite traumatic and results in disturbances to normal sensory, motor, or autonomic function and ultimately impacts a patient's physical, psychological, and social well-being [128]. We already discussed that TQ has activity in spinal cord injury-induced neuropathic pain. Neuroprotective activity of TQ against damages due to trauma-induced both traumatic brain and spinal cord injuries has also reported. In a rat model, after TQ treatment (5 mg/kg, for seven days), neuronal density in contralateral hippocampal regions (CA1, CA2–3, and CA4) significantly increased in comparison to the control group. TQ diminishes the level of MDA in the nuclei and mitochondrial membranes of neurons [129]. Moreover, administration of 30 mg/kg of TQ significantly decreases the histological features of spinal cord damage versus the spinal cord injury group [130].

### 5.2. Protective Activities in Chemical-Induced Neurotoxicity Models

Lead (Pb) is known as a highly neurotoxic agent that causes functional and structural abnormalities in the brains [131]. In addition, Pb induces neurotoxicity in the cerebrum, cerebellum, and medulla oblongata of rat brain. DNA fragmentation and pathological alteration have also evidenced in Pb-intoxicated rat brain [132]. However, TQ protects Pb-induced brain damage. In Sprague-Dawley rats, at a concentration of 0.5 g/l (500 ppm), Pb acetate was given in drinking water. TQ was administered daily at a dose of 20 mg/kg. Regarding brain histology, the control and TQ-treated rats appear normal. Pb acetate treatment results in compromised endothelial lining of the brain blood vessel degeneration with perivascular cuffing of mononuclear cells consistent with lymphocytes, ischemic brain infarction, choroid plexus blood vessel congestion, chromatolysis, and neuronal degeneration and also causes microglial reaction and neuronophagia, hippocampal and cerebellar neuron degeneration, and demyelination of the axon. In contrast, TQ coadministration (20 mg/kg) with Pb acetate markedly reduces the Pb acetate-induced incidence of pathological lesions [133].

Arsenic (As) is capable of crossing the blood-brain barrier and accumulates in different regions of the brain. As exposure is associated with ROS generation, which is supposed to be one of the mechanisms of As-induced oxidative stress [134]. TQ has notable potential effects against As-induced neurotoxicity. Significant and dose-dependent alterations in the level of enzymatic and biochemical biomarkers of oxidative stress were observed in As-treated system. However, pretreatment with TQ (10 *μ*M) significantly reduces As-induced neurotoxicity. Further, a significant decline in As-induced DNA damage was documented with pretreatment of TQ in a comet assay [135]. And also, a recent study reported the ameliorative effect of TQ in As-induced neurotoxicity through antioxidative and antineuroinflammatory mechanisms in female mice. Treatment with arsenate (20 mg/kg) diminishes the levels of norepinephrine, dopamine, AChE, and Na^+^-K^+^-ATPase activities in the cerebral cortex, cerebellum, and brain stem of rats and, likewise, decreases GSH, GSH-Px, CAT, SOD, and GR concentrations. In contrast, As treatment subsequently elevates the levels of 5-hydroxytryptamine, MDA, NO^2−^/nitrate, and TNF-*α*. The presence of degenerated Purkinje cells in the cerebellum was also noticed. Posttreatment with TQ (10 mg/kg) suppresses arsenate-induced neurotoxic effects as decreasing the levels of 5-hydroxytryptamine, MDA, NO, and TNF-*α* and raising the concentrations of norepinephrine, dopamine, GSH, GSH-Px, GR, SOD, and CAT in the cerebral cortex, cerebellum, and brain stem. Likewise, TQ posttreatment increases AChE and Na^+^-K^+^-ATPase activities [136].

A variety of mechanisms has proposed to contribute to ethanol-induced neurotoxicity including excitotoxicity, disruption of cell-cell interactions, apoptosis induction, oxidative stress, and interference with growth factor activity [137]. TQ exhibits protective effects against ethanol-induced neurotoxicity, specifically against apoptosis and cell death. After ethanol (100 mM) exposure for 12 hours, TQ (25 *μ*M) separately and synergistically with metformin (10 mM) improves cell viability and attenuates cytosolic-free calcium [Ca^2+^]_c_ elevation. Additionally, ethanol exposures typically lower the physiological mitochondrial transmembrane potential (Δψ_M_) but metformin and TQ maintain these conditions. In addition, ethanol reduces the expression of Bcl-2 and stimulates the release of cytochrome c from the mitochondria. Through the rise of Bcl-2 expression, metformin and TQ treatment prevents the apoptotic cascade. TQ also suppresses caspase-9 and caspase-3 activation and reduces poly [ADP-ribose] polymerase 1 cleavage. With respect to the relevant staining methods, ethanol treatment confirms more cell death, while metformin and TQ separately or metformin with TQ combined prevents ethanol-induced apoptotic cell death [138].

As a volatile organic compound, toluene is extensively used as an industrial solvent. It is capable of impacting the CNS like depressants, through excitation and psychomotor impairment, followed by inhibition of locomotor activity and sedation. It also brings about oxidative stress, memory loss, and progressive nerve and brain damage [139]. TQ has potential in mediating the effects of toluene-induced neurodegeneration in rat hippocampus and frontal cortex. Chronic toluene exposure causes severe degenerative changes, shrunken cytoplasma, slightly dilated cisternae of the endoplasmic reticulum, markedly swollen mitochondria with degenerated cristae, and nuclear membrane breakdown with chromatin disorganization in neurons of the hippocampus and frontal cortex. TQ (50 mg/kg) treatment protects against such effects [139, 140].

Acrylamide (ACR) is considered a well-documented neurotoxic agent in both humans and laboratory animals, and its existence is a worldwide concern. In addition, subchronic, low-level of occupational human exposure to ACR can lead to neurotoxicity defined by ataxia, skeletal muscle weakness, and numbness of the hands and feet [141]. Oxidative damage has suggested as a mechanism of neurotoxicity induced by ACR [142]. With this, TQ features protective effects in ACR-induced neurotoxicity through preventing oxidative stress. In one experiment, male Wistar rats were treated with ACR (50 mg/kg i.p.) alone or with TQ (2.5, 5, and 10 mg/kg i.p.) for 11 days. Exposure to ACR resulted in severe gait abnormalities while treatment with TQ significantly decreases them. ACR exposures elevate the concentrations of MDA in the cerebral cortex while TQ treatment significantly and dose dependently reduces lipid peroxidation [143].

### 5.3. Radiation-Induced Brain Damage

Through interacting with living cells, ionizing radiation (IR) results in several cytotoxic properties that are mediated through ROS/reactive nitrogen species production. In the brain, TQ modulates IR-induced reactive nitrogen species. One study described a single dose (5 Gy) of total cranial gamma irradiation given to rats and in therapy groups, the dosing of TQ (30 mg/kg/day i.p.) starting 30 min before the radiation dose and after the IR continuing daily for 10 days. In treated rats, *N. sativa* oil or TQ lowers the NO and peroxynitrite levels as well as nitric oxide synthases enzyme activity in brain tissue than in those animals that received ionizing radiation alone [144].

### 5.4. Morphine-Induced Tolerance and Dependence

Repeated administration of morphine and related opiates causes tolerance and dependence [145]. TQ combats morphine-induced tolerance and dependence in mice [44, 146]. In treatment groups, the i.p. doses of TQ (10 mg/kg) administered 30 min before morphine injections continued for up to seven days. TQ inhibits the increase in brain MDA concentrations and NO production, as well as the declines in brain GSH level and GSH-Px activity, induced by repetitive morphine and associated naloxone-induced withdrawal [146]. Furthermore, administration of single or repeated doses of TQ (20 and 40 mg/kg i.p.) significantly decreases the number of jumps in morphine-dependent animals. In addition, TQ attenuates tolerance to the analgesic effect of morphine while high dose of TQ (40 mg/kg) impairs the motor coordination of animals [44].

A summary of TQ's protective effects against chemical-induced neurotoxicity, radiation-induced brain damage, and morphine-induced drug tolerance and dependence is found in Table 3.

### 5.5. Protective Activity in Streptozotocin-Induced Model

In a streptozotocin-induced diabetes model, in the rat's brain, pretreatment with TQ (10 mg/kg p.o.) significantly attenuates increased NO and MDA concentration compared to the control group. In addition, streptozotocin-induced diabetes also significantly decreases GSH and glutathione-s-transferase and CAT; TQ reverses these changes. The increase in GSH and CAT activity with TQ administration may reduce oxidative stress in diabetes mellitus. Moreover, because of chemical nature, TQ reacts with GSH, nicotinamide adenine dinucleotide, and nicotinamide adenine dinucleotide phosphate. Henceforth, a probable intracellular nonenzymatic activation of TQ was presumed dependent upon GSH, nicotinamide adenine dinucleotide, and nicotinamide adenine dinucleotide phosphate representing the “cellular switch” for moderating cellular antioxidant defenses [147].

## 6. Challenges and Delivery Prospects of Thymoquinone in Neurological Disorders

Several clinical trials of TQ have been conducted, but owing to its hydrophobic properties, many phase studies have not been carried out [77]. Thus, TQ clinical studies are a major challenge for researchers. In a phase I trial, it was found safe and well tolerated in patients up to 10 mg/kg/day [148]. In 2010, a study was performed that TQ was administered to a group of children with epilepsy of a sample size of 22 [149]. Regarding this study, TQ had antiepileptic effects in children with refractory seizures [149]. Recently, a study was conducted on sialidosis type 1, an autosomal recessive disorder causing neuraminidase deficiency that causes sialic acid accumulation in tissues. At present, there is no disease-modifying treatment. As a weak histone deacetylase inhibitor, TQ is a Neu 4 sialidase activity agonist and vorinostat is a more potent histone deacetylase inhibitor. However, a clear physiological effect was evidence with TQ on a patient with sialidosis type 1, but not with vorinostat [150].

With respect to the neuropharmacological potential of TQ, it might be a valuable agent against neurological disorders, hence, its formulations and delivery merit investigation. Delivery is also relevant to important to clinical trial studies of TQ against neurological disorders. As per our previous discussion, the chemical nature of TQ is hydrophobic, which causes poor water solubility specifically leading poor formulation characteristics. In human, because of its chemical nature, it has weak membrane penetration [151]. Moreover, TQ has less bioavailability owing to its slow absorption and rapid elimination behavior [60]. Therefore, increasing bioavailability is essential for better pharmacological activities.

In a liver cirrhosis model, encapsulated solid lipid nanoparticles (SLN) of TQ (TQ-SLN) improve oral delivery. With this formulation, an enhanced bioavailability and pharmacodynamics profile could be achieved [152]. In a pharmacokinetic study model, after oral administration to animals, TQ-SLN bioavailability was increased nearly a fivefold. According to the drug distribution analysis, TQ-SLN were found to accumulate more so in the brain than other organs, henceforth, its suitability for brain-targeted drug delivery [153]. Several self-nanoemulsifying drug delivery systems (SNEDDS) of TQ have also been developed and evaluated with regard to oral bioavailability. The oral administration of optimized SNEDDS shows significant improvement in *in vivo* absorption, as well as bioavailability (3.87-fold) of TQ versus TQ suspension [62]. TQ-loaded liposomes and TQ loaded in liposomes modified with Triton X-100 with diameters of roughly 100 nm were found to maintain stability and improve bioavailability [151]. TQ-encapsulated nanoparticles with 97.5% effectiveness in a biodegradable nanoparticulate formulation overcome the poor solubility, thermal and light sensitivity, and minimal systemic bioavailability [154–156]. Additionally, TQ-NLC have been developed to improve bioavailability (elimination half-life~five hours) [157, 158].

A modified form of TQ through chemical conjugation of the quinones with penetrating cations produces neuroprotective activity in a model of middle cerebral artery occlusion. Concerning the study assumption, the mitochondria-targeted delivery of modified TQ significantly increases the drugs' antioxidant efficiency leading to neuroprotective activity against I/R injury [159]. In one investigation of optimized TQ-loaded PLGA-chitosan nanoparticles delivered via nose-to-brain route, TQ elicits neuroprotective activity in a rodent cerebral I/R model. Intranasal delivery of optimized TQ-loaded PLGA-chitosan nanoparticles to the brain significantly reduces the ischemic infarct volume and enhances the locomotor activity and grip strength in the middle cerebral artery-occluded rats. With respect to the biochemical studies, the intranasal delivery of TQ-loaded PLGA-chitosan nanoparticles significantly diminishes lipid peroxidation but elevates antioxidant enzyme activity in the brain. Pharmacokinetic and localization studies showed that TQ-loaded PLGA-chitosan nanoparticles facilitate the delivery of TQ to the brain by an intranasal nose-to-brain transport pathway and enhanced their pharmacokinetic profile in brain tissues [107].

**Figure 2:**
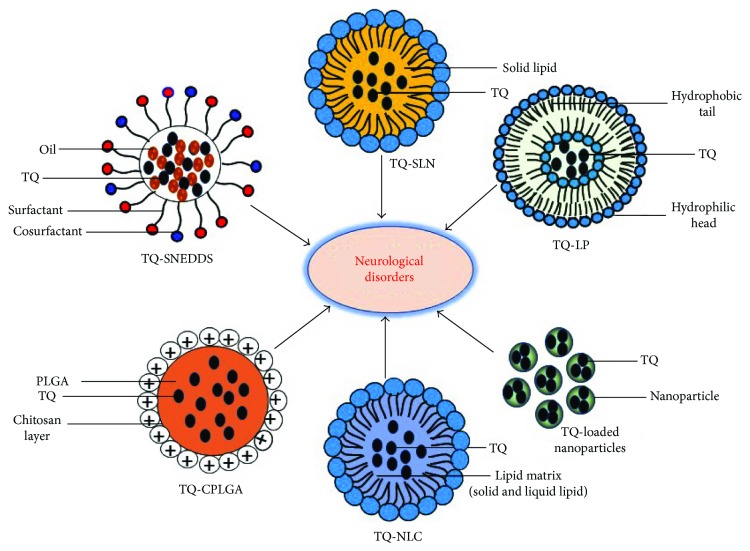
Different formulation of TQ as candidates for neurological disorders. TQ-SLN: TQ solid lipid nanoparticle; TQ-LP: TQ liposome; TQ-SNEDDS: TQ self-nanoemulsifying drug delivery systems; TQ-CPLGA: TQ PLGA-chitosan nanoparticle; TQ-NLC: TQ-loaded nanostructured lipid carrier. It has been proposed that all formulations might have a potential for greater neuropharmacological effects and are probable candidates for treating neurological disorders.

Many novel formulations of TQ have not yet been assessed for neurological disease models. Accordingly, we expect that the aforementioned TQ formulations might be probable candidates for the different neuropharmacological models. As a probable candidate, different formulations of thymoquinone for neurological disorders are portrayed in Figure 2.

## 7. Concluding Remarks

In recent times, phytochemicals have been reported as potential therapeutic alternative candidates for neurological disorders. Regarding the side-effects of modern medicine versus its benefits, the discovery of lesser and/or nontoxic drugs is a principle objective for researchers. Extracts from the various plant parts have potential in various neurological disorders. At the present time, the identification of compounds in the extract responsible for potential activity is also challenging. As a major constituent in *N. sativa*, last few years of research have emphasized research on TQ in various disorders.

In the discussion, by demarcating neurological disorders, we have presented the data concerning the updated pharmacological potential and delivery prospects of TQ. As per our overview, based on neuropharmacological potential, TQ is an indeed an important therapeutic candidate in the treatment of neurological disorders. It features emerging activities in various disease models as well as trauma- and chemical-induced CNS impairments. Further, the effects of TQ on neuroinflammation, neurotoxicity, and neurological disorders, where oxidative stress reduction is a significant effect. Apart from its neuropharmacological activities, the formulation and delivery concepts of TQ as related to neurological disorders are also vital. Henceforth, this study discussed the formulation and delivery prospects of TQ in neurological disorders. This study might be helpful (i) to foster deeper study of TQ in neurological disorders, (ii) to identify the pharmacodynamic profile of TQ, (iii) to discover potential analogs of TQ through various synthetic pathways, and (iv) to design clinical trials to assess TQ in treating neurological disorders. However, many studies have been conducted but further investigations including laboratory-based and bioinformatics approaches are recommended. These investigations might be helpful to establish the precise pharmacological safety, efficacy, and potency profiles of TQ in the therapy for neurological disorders. Overall, in the near future, TQ and its modified forms might contribute in the field of neuromedicine to cure of various neurological diseases and disorders.

## Figures and Tables

**Table 1:** Potential activities of TQ in PD model.

Model	Doses	Mechanistic actions	References
MPP^+^ and rotenone toxicities in dopaminergic neurons	0.01, 0.1, 1, and 10 *μ*M	Increases the number of THir compared with untreated control cultures	[94]
MPP^+^ toxicity in dopaminergic neurons	0.1 and 1 *μ*M	Decreases the number of dopaminergic neurons and increases the release of LDH primary mesencephalic culture by enhancing lysosomal degradation that clears damaged mitochondria and inhibits mitochondria-mediated apoptotic cell death	[95]
6-OHDA-induced PD model	5 and/or 10 mg/kg	Significantly improves turning behavior, prevents loss of SN_PC_ neurons, and lowers the level of MDA	[96]
Rotenone-induced PD model	7.5 and 15 mg/kg	Significantly prevents rotenone-induced motor defects and modifications in the Parkin, Drp1, dopamine, and TH concentrations	[97]

MPP^+^: 1-methyl-4-phenylpyridinium; 6-OHDA: 6-hydroxydopamine; THir: tyrosine hydroxylase immunoreactive; LDH: lactate dehydrogenase; MDA: malondialdehyde; SNpc: substantia nigra pars compacta; Drp1: dynamin-related protein 1; TH: tyrosine hydroxylase.

**Table 2:** Beneficial effects of TQ in epilepsy models.

Model	Doses	Mechanistic actions	References
PTZ-induced epilepsy model	200 and 400 *μ*M	Extends the onset and reduces the tonic-clonic seizure duration	[113]
PTZ- and MES-induced epilepsy model	50 and 100 mg/kg	Potentiates SVP antiepileptic response	[114]
PTZ-induced epilepsy model	20 and 30 mg/kg	TQ and PB combination therapy produces additive anticonvulsant effect	[115]
PTZ-induced epilepsy model	TQ: 40 mg/kg; vitamin C: 250 mg/kg	Activates the GABAB1R/CaMKII/CREB pathway, significantly decreases Bax concentrations, increases Bcl-2 expression, and activates caspase-3	[116]
Penicillin-induced epilepsy model	10, 50, and 100 mg/kg	Prolongs latency time and reduces the spike wave frequency and amplitude of epileptiform activity	[33]
Kainic acid-induced epileptic model	10 mg/kg	Reduces neuronal degeneration (25%) in CA1, CA3, and the dentate hilus; suppresses mossy fiber sprouting (30–40%); treatment also enhances the neurogenesis	[117]
Lithium-pilocarpine rat model	10 mg/kg	Significantly lowered the severity of seizures and significantly elevated Nrf2, HO-1, and SOD expressions	[48]

PTZ: pentylenetetrazole; PB: phenobarbital; SVP: sodium valproate; GABAB1R: gamma-aminobutyric acid B1 receptor; CaMKII: calmodulin-dependent protein kinase II; CREB: cAMP response element-binding protein; MES: maximal electric shock; Bax: Bcl-2-associated X protein; Bcl-2: B-cell lymphoma protein-2; Nrf2: nuclear factor E2-related factor 2; HO-1: heme oxygenase-1; SOD: superoxide dismutase.

**Table 3:** Protective effect of TQ in lead, arsenic, ethanol and toluene-induced neurotoxicity, radiation-induced brain damage, and morphine-induced dependence and tolerance.

Causative agents	Doses of TQ	Potential protective effects	References
Pb	20 mg/kg	Reverses endothelial lining of brain blood vessel degeneration, ischemic brain infarction, choroid plexus blood vessel congestion, chromatolysis and microglial reaction, and neuronophagia; prevents the degeneration of hippocampal and cerebellar neurons and axon demyelination	[133]
As	10 *μ*M, 10 mg/kg, and 20 mg/kg	Alters enzymatic and biochemical markers of oxidative stress; reduces in arsenic-induced DNA damage	[135, 136]
Ethanol	25 *μ*M	Increases Bcl-2 expression, suppresses caspase-9 and caspase-3 activation, and diminishes the PARP-1 cleavage	[138]
Toluene	50 mg/kg	Prevents the degenerative changes, shrunken cytoplasma, slightly dilated cisternae of endoplasmic reticulum, markedly swollen mitochondria with degenerated cristae, and nuclear membrane breakdown with chromatin disorganization in neurons of the hippocampus; also, caspase-3 immunoreactivity	[139, 140]
ACR	2.5, 5, and 10 mg/kg	Protection against oxidative stress	[143]
Radiation	30 mg/kg	In brain tissue, lowers the NO and ONOO (−) levels as well as NOS enzyme activity	[144]
Morphine	10 mg/kg	Reduces oxidative stress markers; prevents tolerance and dependence	[146]

Pb: lead; As: arsenic; ACR: acrylamide; Bcl-2: B-cell lymphoma 2; PARP-1: poly [ADP-ribose] polymerase 1; NO: nitric oxide; ONOO (−): peroxynitrite; NOS: nitric oxide synthases.

## Abbreviations

6-OHDA: 6-Hydroxydopamine
A*β*: Amyloid beta
AD: Alzheimer's disease
AMPK: AMP-activated protein kinase
AMP: Adenosine monophosphate
AChE: Acetylcholine esterase
CAT: Catalase
EAE: Experimental autoimmune encephalomyelitis
GSH: Glutathione
GR: Glutathione reductase
GSH-Px: Glutathione peroxidase
GABA: *γ*-Aminobutyric acid
i.p.: Per intraperitoneal administration
i.c.v: Per intracerebroventricular administration
IC_50_: Half maximal inhibitory concentration
iNOS: Inducible nitric oxide synthase
I/R: Ischemia/reperfusion
IL: Interleukin
LD_50_: Median lethal dose
LPS: Lipopolysaccharide
LDH: Lactate dehydrogenase
MDA: Malondialdehyde
MPP^+^: 1-Methyl-4-phenylpyridinium ion
NF-*κ*B: Nuclear factor kappa-light-chain-enhancer of activated B cells
NO: Nitric oxide
NO^2−^: Nitrite
ONOO (−): Peroxynitrite
PD: Parkinson's disease
PTZ: Pentylenetetrazole
p.o.: Per oral administration
ROS: Reactive oxygen species
SOD: Superoxide dismutase
SE: Status epilepticus
SIRT1: Sirtuin 1
TQ: Thymoquinone
TNF-*α*: Tumor necrosis factor-*α*
TBARS: Thiobarbituric acid reactive substance
THir: Tyrosine hydroxylase immunoreactive.

## References

[1] Ahmad Z., Laughlin T. F., Kady I. O. (2015). Thymoquinone inhibits *Escherichia coli* ATP synthase and cell growth. *PLoS One*.

[2] Gray J. P., Burgos D. Z., Yuan T. (2016). Thymoquinone, a bioactive component of *Nigella sativa*, normalizes insulin secretion from pancreatic *β*-cells under glucose overload via regulation of malonyl-CoA. *American Journal of Physiology-Endocrinology and Metabolism*.

[3] Hossain M. K., Basak D., Sayeed B., Shahdaat M. (2017). Thymoquinone as a potential complementary adjuvant therapy for cancer treatment: evidence from preclinical studies. *Frontiers in Pharmacology*.

[4] Hajhashemi V., Ghannadi A., Jafarabadi H. (2004). Black cumin seed essential oil, as a potent analgesic and antiinflammatory drug. *Phytotherapy Research*.

[5] Ahmad A., Husain A., Mujeeb M. (2013). A review on therapeutic potential of *Nigella sativa*: a miracle herb. *Asian Pacific Journal Tropical Biomedicine*.

[6] Burits M., Bucar F. (2000). Antioxidant activity of *Nigella sativa* essential oil. *Phytotherapy Research*.

[7] Salem M. L. (2005). Immunomodulatory and therapeutic properties of the *Nigella sativa* L. seed. *International Immunopharmacology*.

[8] Butt M. S., Sultan M. T. (2010). *Nigella sativa*: reduces the risk of various maladies. *Critical Reviews in Food Science and Nutrition*.

[9] Hosseinzadeh H., Eskandari M., Ziaee T. (2008). Antitussive effect of thymoquinone, a constituent of *Nigella sativa* seeds, in guinea pigs. *Pharmacology*.

[10] Kanter M., Demir H., Karakaya C., Ozbek H. (2005). Gastroprotective activity of *Nigella sativa* L oil and its constituent, thymoquinone against acute alcohol-induced gastric mucosal injury in rats. *World Journal of Gastroenterology*.

[11] Amin B., Hosseinzadeh H. (2016). Black cumin (*Nigella sativa*) and its active constituent, thymoquinone: an overview on the analgesic and anti-inflammatory effects. *Planta Medica*.

[12] Chehl N., Chipitsyna G., Gong Q., Yeo C. J., Arafat H. A. (2009). Anti-inflammatory effects of the *Nigella sativa* seed extract, thymoquinone, in pancreatic cancer cells. *HPB*.

[13] Inci M., Davarci M., Inci M. (2012). Anti-inflammatory and antioxidant activity of thymoquinone in a rat model of acute bacterial prostatitis. *Human & Experimental Toxicology*.

[14] Kanter M., Coskun O., Uysal H. (2006). The antioxidative and antihistaminic effect of *Nigella sativa* and its major constituent, thymoquinone on ethanol-induced gastric mucosal damage. *Archives of Toxicology*.

[15] Ullah R., Rehman A., Zafeer M. F. (2017). Anthelmintic potential of thymoquinone and curcumin on *Fasciola gigantica*. *PLoS One*.

[16] Chaieb K., Kouidhi B., Jrah H., Mahdouani K., Bakhrouf A. (2011). Antibacterial activity of thymoquinone, an active principle of *Nigella sativa* and its potency to prevent bacterial biofilm formation. *BMC Complementary and Alternative Medicine*.

[17] Randhawa M. A., Alenazy A. K., Alrowaili M. G., Basha J. (2017). An active principle of *Nigella sativa* L., thymoquinone, showed significant antimicrobial activity against anaerobic bacteria. *Journal of Intercultural Ethnopharmacology*.

[18] Solati Z., Baharin B. S., Bagheri H. (2014). Antioxidant property, thymoquinone content and chemical characteristics of different extracts from *Nigella sativa* L. seeds. *Journal of the American Oil Chemists' Society*.

[19] Ince S., Kucukkurt I., Demirel H. H., Turkmen R., Zemheri F., Akbel E. (2013). The role of thymoquinone as antioxidant protection on oxidative stress induced by imidacloprid in male and female Swiss albino mice. *Toxicological & Environmental Chemistry*.

[20] Gholamnezhad Z., Rafatpanah H., Sadeghnia H. R., Boskabady M. H. (2015). Immunomodulatory and cytotoxic effects of *Nigella sativa* and thymoquinone on rat splenocytes. *Food and Chemical Toxicology*.

[21] Attoub S., Sperandio O., Raza H. (2013). Thymoquinone as an anticancer agent: evidence from inhibition of cancer cells viability and invasion *in vitro* and tumor growth *in vivo*. *Fundamental & Clinical Pharmacology*.

[22] Majdalawieh A. F., Fayyad M. W., Nasrallah G. K. (2017). Anti-cancer properties and mechanisms of action of thymoquinone, the major active ingredient of *Nigella sativa*. *Critical Reviews in Food Science and Nutrition*.

[23] Suddek G. M. (2014). Protective role of thymoquinone against liver damage induced by tamoxifen in female rats. *Canadian Journal of Physiology and Pharmacology*.

[24] Gonca E., Kurt Ç. (2015). Cardioprotective effect of thymoquinone: a constituent of *Nigella sativa* L., against myocardial ischemia/reperfusion injury and ventricular arrhythmias in anaesthetized rats. *Pakistan Journal of Pharmaceutical Sciences*.

[25] Ojha S., Azimullah S., Mohanraj R. (2015). Thymoquinone protects against myocardial ischemic injury by mitigating oxidative stress and inflammation. *Evidence-based Complementary and Alternative Medicine*.

[26] Alimohammadi S., Hobbenaghi R., Javanbakht J. (2013). RETRACTED ARTICLE: Protective and antidiabetic effects of extract from *Nigella sativa* on blood glucose concentrations against streptozotocin (STZ)-induced diabetic in rats: an experimental study with histopathological evaluation. *Diagnostic Pathology*.

[27] El-Ameen N. M. H., Taha M. M. E., Abdelwahab S. I. (2015). Anti-diabetic properties of thymoquinone is unassociated with glycogen phosphorylase inhibition. *Pharmacognosy Journal*.

[28] Sagit M., Korkmaz F., Akcadag A., Somdas M. A. (2013). Protective effect of thymoquinone against cisplatin-induced ototoxicity. *European Archives of Oto-Rhino-Laryngology*.

[29] Aksoy F., Dogan R., Ozturan O. (2015). An evaluation of the protective effects of thymoquinone on amikacin-induced ototoxicity in rats. *Clinical and Experimental Otorhinolaryngology*.

[30] Badary O. A., Nagi M. N., Al-Shabanah O. A., Al-Sawaf H. A., Al-Sohaibani M. O., Al-Bekairi A. M. (1997). Thymoquinone ameliorates the nephrotoxicity induced by cisplatin in rodents and potentiates its antitumor activity. *Canadian Journal of Physiology and Pharmacology*.

[31] Ustyol L., Demirören K., Kandemir I. (2016). Comparative nephroprotective effects of silymarin, N-acetylcysteine, and thymoquinone against carbon tetrachloride-induced nephrotoxicity in rats. *Iranian Red Crescent Medical Journal*.

[32] Hosseinzadeh H., Parvardeh S. (2004). Anticonvulsant effects of thymoquinone, the major constituent of *Nigella sativa* seeds, in mice. *Phytomedicine*.

[33] Beyazcicek E., Ankarali S., Beyazcicek O., Ankarali H., Demir S., Ozmerdivenli R. (2016). Effects of thymoquinone, the major constituent of *Nigella sativa* seeds, on penicillin-induced epileptiform activity in rats. *Neurosciences*.

[34] Bano F., Ahmed A., Parveen T., Haider S. (2014). Anxiolytic and hyperlocomotive effects of aqueous extract of *Nigella sativa* L. seeds in rats. *Pakistan Journal of Pharmaceutical Sciences*.

[35] Perveen T., Haider S., Kanwal S., Haleem D. J. (2009). Repeated administration of *Nigella sativa* decreases 5-HT turnover and produces anxiolytic effects in rats. *Pakistan Journal of Pharmaceutical Sciences*.

[36] Gilhotra N., Dhingra D. (2011). Thymoquinone produced antianxiety-like effects in mice through modulation of GABA and NO levels. *Pharmacological Reports*.

[37] Perveen T., Haider S., Zuberi N. A., Saleem S., Sadaf S., Batool Z. (2014). Increased 5-HT levels following repeated administration of *Nigella sativa* L.(black seed) oil produce antidepressant effects in rats. *Scientia Pharmaceutica*.

[38] Elkhayat E. S., Alorainy M. S., El-Ashmawy I. M., Fat’hi S. (2016). Potential antidepressant constituents of *Nigella sativa* seeds. *Pharmacognosy Magazine*.

[39] Aquib M., Najmi A., Akhtar M. (2015). Antidepressant effect of thymoquinone in animal models of depression. *Drug Research*.

[40] Khan R. A., Najmi A. K., Khuroo A. H., Goswami D., Akhtar M. (2014). Ameliorating effects of thymoquinone in rodent models of schizophrenia. *African Journal of Pharmacy and Pharmacology*.

[41] El-Marasy S. A., El-Shenawy S. M., El-Khatib A. S., El-Shabrawy O. A., Kenawy S. A. (2012). Effect of *Nigella sativa* and wheat germ oils on scopolamine-induced memory impairment in rats. *Bulletin of Faculty of Pharmacy, Cairo University*.

[42] Sayeed M. S. B., Asaduzzaman M., Morshed H., Hossain M. M., Kadir M. F., Rahman M. R. (2013). The effect of *Nigella sativa* Linn. seed on memory, attention and cognition in healthy human volunteers. *Journal of Ethnopharmacology*.

[43] Sahak M. K. A., Kabir N., Abbas G., Draman S., Hashim N. H., Hasan Adli D. S. (2016). The role of *Nigella sativa* and its active constituents in learning and memory. *Evidence-based Complementary and Alternative Medicine*.

[44] Hosseinzadeh H., Parvardeh S., Masoudi A., Moghimi M., Mahboobifard F. (2016). Attenuation of morphine tolerance and dependence by thymoquinone in mice. *Avicenna Journal of Phytomedicine*.

[45] Mousavi S., Tayarani-Najaran Z., Asghari M., Sadeghnia H. (2010). Protective effect of *Nigella sativa* extract and thymoquinone on serum/glucose deprivation-induced PC12 cells death. *Cellular and Molecular Neurobiology*.

[46] Sandhu K. S., Rana A. C. (2013). Evaluation of anti Parkinson’s activity of *Nigella sativa* (kalonji) seeds in chlorpromazine induced experimental animal model. *International Journal of Pharmacy and Pharmaceutical Sciences*.

[47] Jahromy M. H., Jalili M., Mohajer A. J., Poor F. K., Dara S. M. (2014). Effects of *Nigella sativa* seed extract on perphenzine-induced muscle rigidity in male mice. *World Journal of Neuroscience*.

[48] Shao Y.-y., Li B., Huang Y.-m., Luo Q., Xie Y.-m., Chen Y.-h. (2017). Thymoquinone attenuates brain injury via an antioxidative pathway in a status epilepticus rat model. *Translational Neuroscience*.

[49] Velagapudi R., El-Bakoush A., Lepiarz I., Ogunrinade F., Olajide O. A. (2017). AMPK and SIRT1 activation contribute to inhibition of neuroinflammation by thymoquinone in BV2 microglia. *Molecular and Cellular Biochemistry*.

[50] Gökce E. C., Kahveci R., Gökce A. (2016). Neuroprotective effects of thymoquinone against spinal cord ischemia-reperfusion injury by attenuation of inflammation, oxidative stress, and apoptosis. *Journal of Neurosurgery: Spine*.

[51] Chen L., Li B., Chen B. (2016). Thymoquinone alleviates the experimental diabetic peripheral neuropathy by modulation of inflammation. *Scientific Reports*.

[52] September 2017, https://pubchem.ncbi.nlm.nih.gov/compound/Thymoquinone#section

[53] Ecevit H., Gunduz K., Bilgic N., Izmirli M., Gogebakan B. (2017). The effect of thymoquinone on BEAS-2B cell viability and TGF-*β*1 release. *Advances in Modern Oncology Research*.

[54] Mihara S., Shibamoto T. (2015). The role of flavor and fragrance chemicals in TRPA1 (transient receptor potential cation channel, member A1) activity associated with allergies. *Allergy, Asthma & Clinical Immunology*.

[55] Arnott J. A., Planey S. L. (2012). The influence of lipophilicity in drug discovery and design. *Expert Opinion on Drug Discovery*.

[56] Trippier P. C. (2016). Selecting good ‘drug-like’ properties to optimize small molecule blood-brain barrier penetration. *Current Medicinal Chemistry*.

[57] Mabrouk A., Cheikh H. B. (2016). Thymoquinone ameliorates lead-induced suppression of the antioxidant system in rat kidneys. *Libyan Journal of Medicine*.

[58] Dockal E. R., Cass Q. B., Brocksom T. J., Brocksom U., Corrěa A. G. (2006). A simple and efficient synthesis of thymoquinone and methyl p-benzoquinone. *Synthetic Communications*.

[59] Alkharfy K. M., Ahmad A., Khan R. M., Al-Asmari M. (2013). High-performance liquid chromatography of thymoquinone in rabbit plasma and its application to pharmacokinetics. *Journal of Liquid Chromatography & Related Technologies*.

[60] Alkharfy K. M., Ahmad A., Khan R. M., Al-Shagha W. M. (2015). Pharmacokinetic plasma behaviors of intravenous and oral bioavailability of thymoquinone in a rabbit model. *European Journal of Drug Metabolism and Pharmacokinetics*.

[61] Elmowafy M., Samy A., Raslan M. A. (2016). Enhancement of bioavailability and pharmacodynamic effects of thymoquinone via nanostructured lipid carrier (NLC) formulation. *AAPS PharmSciTech*.

[62] Kalam M. A., Raish M., Ahmed A. (2017). Oral bioavailability enhancement and hepatoprotective effects of thymoquinone by self-nanoemulsifying drug delivery system. *Materials Science and Engineering: C*.

[63] Alkharfy K. M. (2014). Pharmacokinetics and pharmacodynamics of thymoquinone as a novel agent in sepsis management. *Journal of Bioequivalence & Bioavailability*.

[64] Ahmad A., Khan R., Alkharfy K. M., Raish M., Al-Jenoobi F. I., Al-Mohizea A. M. (2015). Effects of thymoquinone on the pharmacokinetics and pharmacodynamics of glibenclamide in a rat model. *Natural Product Communications*.

[65] Lupidi G., Camaioni E., Khalifé H. (2012). Characterization of thymoquinone binding to human *α*_1_-acid glycoprotein. *Journal of Pharmaceutical Sciences*.

[66] Lupidi G., Scire A., Camaioni E. (2010). Thymoquinone, a potential therapeutic agent of *Nigella sativa*, binds to site I of human serum albumin. *Phytomedicine*.

[67] El-Najjar N., Ketola R. A., Nissilä T. (2011). Impact of protein binding on the analytical detectability and anticancer activity of thymoquinone. *Journal of Chemical Biology*.

[68] El-Najjar N., Ketola R., Urtti A., Gali-Muhtaseb H., Vuorela H. (2010). Impact of protein binding on thymoquinone's analytical detection. *Planta Medica*.

[69] Nagi M. N., Almakki H. A. (2009). Thymoquinone supplementation induces quinone reductase and glutathione transferase in mice liver: possible role in protection against chemical carcinogenesis and toxicity. *Phytotherapy Research*.

[70] Khalife K., Lupidi G. (2009). Nonenzymatic reduction of thymoquinone in physiological conditions. *Free Radical Research*.

[71] Qadri S. M., Mahmud H., Föller M., Lang F. (2009). Thymoquinone-induced suicidal erythrocyte death. *Food and Chemical Toxicology*.

[72] Khader M., Bresgen N., Eckl P. (2009). *In vitro* toxicological properties of thymoquinone. *Food and Chemical Toxicology*.

[73] Al-Ali A., Alkhawajah A. A., Randhawa M. A., Shaikh N. A. (2008). Oral and intraperitoneal LD_50_ of thymoquinone, an active principle of *Nigella sativa*, in mice and rats. *Journal of Ayub Medical College, Abbottabad*.

[74] Mansour M., Ginawi O., El-Hadiyah T., El-Khatib A., Al-Shabanah O., Al-Sawaf H. (2001). Effects of volatile oil constituents of *Nigella sativa* on carbon tetrachloride-induced hepatotoxicity in mice: evidence for antioxidant effects of thymoquinone. *Research Communications in Molecular Pathology and Pharmacology*.

[75] Alam M., Galav V. (2013). Subacute 28 days repeated toxicity assessment of thymoquinone (volatile oil of black seed) in Wistar rats. *Indian Journal of Scientific Research*.

[76] Tubesha Z., Imam M. U., Mahmud R., Ismail M. (2013). Study on the potential toxicity of a thymoquinone-rich fraction nanoemulsion in Sprague Dawley rats. *Molecules*.

[77] Ong Y. S., Yazan L. S., Ng W. K. (2016). Acute and subacute toxicity profiles of thymoquinone-loaded nanostructured lipid carrier in BALB/c mice. *International Journal of Nanomedicine*.

[78] Velho-Pereira R., Kumar A., Pandey B. N., Jagtap A. G., Mishra K. P. (2011). Radiosensitization in human breast carcinoma cells by thymoquinone: role of cell cycle and apoptosis. *Cell Biology International*.

[79] Dirican A., Sahin O., Tasli F. (2016). Thymoquinone enhances cisplatin-induced neprotoxicity in high dose. *Journal of Oncological Science*.

[80] Morales I., Guzmán-Martínez L., Cerda-Troncoso C., Farías G. A., Maccioni R. B. (2014). Neuroinflammation in the pathogenesis of Alzheimer’s disease. A rational framework for the search of novel therapeutic approaches. *Frontiers in Cellular Neuroscience*.

[81] Taka E., Mazzio E. A., Goodman C. B. (2015). Anti-inflammatory effects of thymoquinone in activated BV-2 microglial cells. *Journal of Neuroimmunology*.

[82] Velagapudi R., Kumar A., Bhatia H. S. (2017). Inhibition of neuroinflammation by thymoquinone requires activation of Nrf2/ARE signalling. *International Immunopharmacology*.

[83] Hossen M. J., Yang W. S., Kim D., Aravinthan A., Kim J.-H., Cho J. Y. (2017). Thymoquinone: an IRAK1 inhibitor with *in vivo* and *in vitro* anti-inflammatory activities. *Scientific Reports*.

[84] Bargi R., Asgharzadeh F., Beheshti F., Hosseini M., Sadeghnia H. R., Khazaei M. (2017). The effects of thymoquinone on hippocampal cytokine level, brain oxidative stress status and memory deficits induced by lipopolysaccharide in rats. *Cytokine*.

[85] Garcez M. L., Mina F., Bellettini-Santos T. (2017). Minocycline reduces inflammatory parameters in the brain structures and serum and reverses memory impairment caused by the administration of amyloid *β* (1-42) in mice. *Progress in Neuro-Psychopharmacology and Biological Psychiatry*.

[86] Annicchiarico R., Federici A., Pettenati C., Caltagirone C. (2007). Rivastigmine in Alzheimer’s disease: cognitive function and quality of life. *Therapeutics and Clinical Risk Management*.

[87] Ismail Z., Nguyen M.-Q., Fischer C. E., Schweizer T. A., Mulsant B. H., Mamo D. (2011). Neurobiology of delusions in Alzheimer’s disease. *Current Psychiatry Reports*.

[88] Forner S., Baglietto-Vargas D., Martini A. C., Trujillo-Estrada L., LaFerla F. M. (2017). Synaptic impairment in Alzheimer’s disease: a dysregulated symphony. *Trends in Neurosciences*.

[89] Alhebshi A., Gotoh M., Suzuki I. (2013). Thymoquinone protects cultured rat primary neurons against amyloid *β*-induced neurotoxicity. *Biochemical and Biophysical Research Communications*.

[90] Norsharina I., Maznah I., Iqbal S., Latiff L. A. (2013). Anti-aggregation effects of thymoquinone against Alzheimer’s *β*-amyloid in vitro. *Journal of Medicinal Plants Research*.

[91] Fattah L. I. A., Zickri M. B., Aal L. A., Heikal O., Osama E. (2016). The effect of thymoquinone, *α*7 receptor agonist and *α*7 receptor allosteric modulator on the cerebral cortex in experimentally induced Alzheimer’s disease in relation to MSCs activation. *International Journal of Stem Cells*.

[92] More S., Choi D.-K. (2017). Neuroprotective role of atractylenolide-I in an *in vitro* and *in vivo* model of Parkinson’s disease. *Nutrients*.

[93] Thomas B. (2009). Parkinson’s disease: from molecular pathways in disease to therapeutic approaches. *Antioxidants & Redox Signaling*.

[94] Radad K., Moldzio R., Taha M., Rausch W. D. (2009). Thymoquinone protects dopaminergic neurons against MPP^+^ and rotenone. *Phytotherapy Research*.

[95] Radad K. S., Al-Shraim M. M., Moustafa M. F., Rausch W.-D. (2015). Neuroprotective role of thymoquinone against 1-methyl-4-phenylpyridinium-induced dopaminergic cell death in primary mesencephalic cell culture. *Neurosciences*.

[96] Sedaghat R., Roghani M., Khalili M. (2014). Neuroprotective effect of thymoquinone, the *Nigella sativa* bioactive compound, in 6-hydroxydopamine-induced hemi-parkinsonian rat model. *Iranian Journal of Pharmaceutical Research*.

[97] Ebrahimi S. S., Oryan S., Izadpanah E., Hassanzadeh K. (2017). Thymoquinone exerts neuroprotective effect in animal model of Parkinson’s disease. *Toxicology Letters*.

[98] Zeng Y., Song C., Ding X., Ji X., Yi L., Zhu K. (2007). Baicalin reduces the severity of experimental autoimmune encephalomyelitis. *Brazilian Journal of Medical and Biological Research*.

[99] Miljković D., Dekanski D., Miljković Ž., Momčilović M., Mostarica-Stojkovic M. (2009). Dry olive leaf extract ameliorates experimental autoimmune encephalomyelitis. *Clinical Nutrition*.

[100] Mohamed A., Shoker A., Bendjelloul F. (2003). Improvement of experimental allergic encephalomyelitis (EAE) by thymoquinone; an oxidative stress inhibitor. *Biomedical Sciences Instrumentation*.

[101] Mohamed A., Afridi D., Garani O., Tucci M. (2004). Thymoquinone inhibits the activation of NF-kappaB in the brain and spinal cord of experimental autoimmune encephalomyelitis. *Biomedical Sciences Instrumentation*.

[102] Mohamed A., Waris H., Ramadan H., Quereshi M., Kalra J. (2009). Amelioration of chronic relapsing experimental autoimmune encephalomyelitis (cr-eae) using thymoquinone-biomed 2009. *Biomedical Sciences Instrumentation*.

[103] Yao Y., Chen L., Xiao J. (2014). Chrysin protects against focal cerebral ischemia/reperfusion injury in mice through attenuation of oxidative stress and inflammation. *International Journal of Molecular Sciences*.

[104] Ahmed M. A., El Morsy E. M., Ahmed A. A. (2014). Pomegranate extract protects against cerebral ischemia/reperfusion injury and preserves brain DNA integrity in rats. *Life Sciences*.

[105] Al-Majed A. A., Al-Omar F. A., Nagi M. N. (2006). Neuroprotective effects of thymoquinone against transient forebrain ischemia in the rat hippocampus. *European Journal of Pharmacology*.

[106] Ahmad N., Ahmad R., Alam M. A., Samim M., Iqbal Z., Ahmad F. J. (2016). Quantification and evaluation of thymoquinone loaded mucoadhesive nanoemulsion for treatment of cerebral ischemia. *International Journal of Biological Macromolecules*.

[107] Xiao X.-Y., Zhu Y.-X., Bu J.-Y., Li G.-W., Zhou J.-H., Zhou S.-P. (2016). Evaluation of neuroprotective effect of thymoquinone nanoformulation in the rodent cerebral ischemia-reperfusion model. *BioMed Research International*.

[108] Elvevag B., Goldberg T. E. (2000). Cognitive impairment in schizophrenia is the core of the disorder. *Critical Reviews in Neurobiology*.

[109] Dey A., Das S., Mukherjee A. (2016). Possible natural therapeutics against schizophrenia and its acute and treatment resistant forms: a review. *Journal of Biologically Active Products from Nature*.

[110] Chatterjee M., Verma R., Kumari R. (2015). Antipsychotic activity of standardized *Bacopa* extract against ketamine-induced experimental psychosis in mice: evidence for the involvement of dopaminergic, serotonergic, and cholinergic systems. *Pharmaceutical Biology*.

[111] Govindu S., Adikay S. (2014). Evaluation of antiepileptic activity of chloroform extract of *Acalypha fruticosa* in mice. *Pharmacognosy Research*.

[112] Abdollahi Fard M., Shojaii A. (2013). Efficacy of Iranian traditional medicine in the treatment of epilepsy. *BioMed Research International*.

[113] Hosseinzadeh H., Parvardeh S., Nassiri-Asl M., Mansouri M.-T. (2005). Intracerebroventricular administration of thymoquinone, the major constituent of *Nigella sativa* seeds, suppresses epileptic seizures in rats. *Medical Science Monitor*.

[114] Raza M., Alghasham A. A., Alorainy M. S., El-Hadiyah T. M. (2006). Beneficial interaction of thymoquinone and sodium valproate in experimental models of epilepsy: reduction in hepatotoxicity of valproate. *Scientia Pharmaceutica*.

[115] Mostafa R. M., Moustafa Y. M., Mirghani Z. (2012). Thymoquinone alone or in combination with phenobarbital reduces the seizure score and the oxidative burden in pentylenetetrazole-kindled rats. *Oxidants and Antioxidants in Medical Science*.

[116] Ullah I., Badshah H., Naseer M. I., Lee H. Y., Kim M. O. (2015). Thymoquinone and vitamin C attenuates pentylenetetrazole-induced seizures via activation of GABAB1 receptor in adult rats cortex and hippocampus. *Neuromolecular Medicine*.

[117] Brito S. A., Rao M. S. (2016). Thymoquinone enhances neurogenesis to a greater extent in middle-aged than in young aged rat in chronic epilepsy. *The FASEB Journal*.

[118] Alhamdan A. A. (2013). Neuroprotective effect of thymoquinone on repeated immobilization stress-induced oxidative stress in rats. *Asian Journal of Medical Sciences*.

[119] Julius D., Basbaum A. I. (2001). Molecular mechanisms of nociception. *Nature*.

[120] Alemy S., Karami M., Hossini E., Ebrahimzadeh M., Majd N. S. (2012). Antinociceptive activity and effect of methanol extract of *Salvia limbata* on withdrawal syndrome in mice. *European Review for Medical and Pharmacological Sciences*.

[121] Sayeed M. A., Ali M. H. (2015). Investigations of analgesic activity of the methanol extract of *Haldina cordifolia* (Roxb.) bark by using *in vivo* animal model studies. *Research Journal of Botany*.

[122] Costigan M., Scholz J., Woolf C. J. (2009). Neuropathic pain: a maladaptive response of the nervous system to damage. *Annual Review of Neuroscience*.

[123] Amin B., Taheri M. M. H., Hosseinzadeh H. (2014). Effects of intraperitoneal thymoquinone on chronic neuropathic pain in rats. *Planta Medica*.

[124] Çelik F., Göçmez C., Karaman H. (2014). Therapeutic effects of thymoquinone in a model of neuropathic pain. *Current Therapeutic Research*.

[125] de Sousa D. P., Nóbrega F. F., Santos C. C. (2012). Antinociceptive activity of thymoquinone and its structural analogues: a structure-activity relationship study. *Tropical Journal of Pharmaceutical Research*.

[126] Loane D. J., Stoica B. A., Faden A. I. (2015). Neuroprotection for traumatic brain injury. *Handbook of Clinical Neurology*.

[127] Xing Z., Xia Z., Peng W. (2016). Xuefu Zhuyu decoction, a traditional Chinese medicine, provides neuroprotection in a rat model of traumatic brain injury via an anti-inflammatory pathway. *Scientific Reports*.

[128] Singh A., Tetreault L., Kalsi-Ryan S., Nouri A., Fehlings M. G. (2014). Global prevalence and incidence of traumatic spinal cord injury. *Clinical Epidemiology*.

[129] Gülşen İ., Ak H., Çölçimen N. (2016). Neuroprotective effects of thymoquinone on the hippocampus in a rat model of traumatic brain injury. *World Neurosurgery*.

[130] Üstün N., Aras M., Ozgur T. (2014). Thymoquinone attenuates trauma induced spinal cord damage in an animal model. *Ulusal Travma ve Acil Cerrahi Dergisi*.

[131] Phyu M. P., Tangpong J. (2013). Protective effect of *Thunbergia laurifolia* (Linn.) on lead induced acetylcholinesterase dysfunction and cognitive impairment in mice. *BioMed Research International*.

[132] Khalaf A., Moselhy W. A., Abdel-Hamed M. I. (2012). The protective effect of green tea extract on lead induced oxidative and DNA damage on rat brain. *Neurotoxicology*.

[133] Radad K., Hassanein K., Al-Shraim M., Moldzio R., Rausch W.-D. (2014). Thymoquinone ameliorates lead-induced brain damage in Sprague Dawley rats. *Experimental and Toxicologic Pathology*.

[134] Prakash C., Soni M., Kumar V. (2016). Mitochondrial oxidative stress and dysfunction in arsenic neurotoxicity: a review. *Journal of Applied Toxicology*.

[135] Firdaus F., Zafeer M. F., Anis E., Fatima M., Hossain M. M., Afzal M. (2016). Antioxidant potential of thymoquinone against arsenic mediated neurotoxicity. *Free Radicals and Antioxidants*.

[136] Kassab R. B., El-Hennamy R. E. (2017). The role of thymoquinone as a potent antioxidant in ameliorating the neurotoxic effect of sodium arsenate in female rat. *Egyptian Journal of Basic and Applied Sciences*.

[137] Goodlett C. R., Horn K. H., Zhou F. C. (2005). Alcohol teratogenesis: mechanisms of damage and strategies for intervention. *Experimental Biology and Medicine*.

[138] Ullah I., Ullah N., Naseer M. I., Lee H. Y., Kim M. O. (2012). Neuroprotection with metformin and thymoquinone against ethanol-induced apoptotic neurodegeneration in prenatal rat cortical neurons. *BMC Neuroscience*.

[139] Kanter M. (2011). Protective effects of thymoquinone on the neuronal injury in frontal cortex after chronic toluene exposure. *Journal of Molecular Histology*.

[140] Kanter M. (2008). *Nigella sativa* and derived thymoquinone prevents hippocampal neurodegeneration after chronic toluene exposure in rats. *Neurochemical Research*.

[141] Pan X., Zhu L., Lu H., Wang D., Lu Q., Yan H. (2015). Melatonin attenuates oxidative damage induced by acrylamide in vitro and in vivo. *Oxidative Medicine and Cellular Longevity*.

[142] Prasad S. N. (2012). Evidence of acrylamide induced oxidative stress and neurotoxicity in *Drosophila melanogaster*–its amelioration with spice active enrichment: relevance to neuropathy. *Neurotoxicology*.

[143] Mehri S., Shahi M., Razavi B. M., Hassani F. V., Hosseinzadeh H. (2014). Neuroprotective effect of thymoquinone in acrylamide-induced neurotoxicity in Wistar rats. *Iranian Journal of Basic Medical Sciences*.

[144] Ahlatci A., Kuzhan A., Taysi S. (2014). Radiation-modifying abilities of *Nigella sativa* and thymoquinone on radiation-induced nitrosative stress in the brain tissue. *Phytomedicine*.

[145] Williams C. L., Bihm C. C., Rosenfeld G. C., Burks T. F. (1997). Morphine tolerance and dependence in the rat intestine *in vivo*. *Journal of Pharmacology and Experimental Therapeutics*.

[146] Abdel-Zaher A. O., Mostafa M. G., Farghly H. M., Hamdy M. M., Omran G. A., Al-Shaibani N. K. (2013). Inhibition of brain oxidative stress and inducible nitric oxide synthase expression by thymoquinone attenuates the development of morphine tolerance and dependence in mice. *European Journal of Pharmacology*.

[147] Hamdy N. M., Taha R. A. (2009). Effects of *Nigella sativa* oil and thymoquinone on oxidative stress and neuropathy in streptozotocin-induced diabetic rats. *Pharmacology*.

[148] Al-Amri A. M., Bamosa A. O. (2009). Phase I safety and clinical activity study of thymoquinone in patients with advanced refractory malignant disease. *Shiraz E-Medical Journal*.

[149] Akhondian J., Kianifar H., Raoofziaee M., Moayedpour A., Toosi M. B., Khajedaluee M. (2011). The effect of thymoquinone on intractable pediatric seizures (pilot study). *Epilepsy Research*.

[150] Liang C., Peach D., Stark S., Fietz M. (2017). N-of-1 trial of thymoquinone and vorinostat in a patient with sialidosis type 1. *Journal of Neurology, Neurosurgery & Psychiatry*.

[151] Odeh F., Ismail S. I., Abu-Dahab R., Mahmoud I. S., Al Bawab A. (2012). Thymoquinone in liposomes: a study of loading efficiency and biological activity towards breast cancer. *Drug Delivery*.

[152] Singh A., Ahmad I., Akhter S. (2013). Nanocarrier based formulation of thymoquinone improves oral delivery: stability assessment, *in vitro* and *in vivo* studies. *Colloids and Surfaces B: Biointerfaces*.

[153] Surekha R., Sumathi T. (2016). An efficient encapsulation of thymoquinone using solid lipid nanoparticle for brain targeted drug delivery: physicochemical characterization, pharmacokinetics and bio-distribution studies. *International Journal of Pharmaceutical and Clinical Research*.

[154] Ravindran J., Nair H. B., Sung B., Prasad S., Tekmal R. R., Aggarwal B. B. (2010). Thymoquinone poly (lactide-co-glycolide) nanoparticles exhibit enhanced anti-proliferative, anti-inflammatory, and chemosensitization potential. *Biochemical Pharmacology*.

[155] Bhattacharya S., Ahir M., Patra P. (2015). PEGylated-thymoquinone-nanoparticle mediated retardation of breast cancer cell migration by deregulation of cytoskeletal actin polymerization through miR-34a. *Biomaterials*.

[156] Nair H. B., Sung B., Yadav V. R., Kannappan R., Chaturvedi M. M., Aggarwal B. B. (2010). Delivery of antiinflammatory nutraceuticals by nanoparticles for the prevention and treatment of cancer. *Biochemical Pharmacology*.

[157] Ng W. K., Saiful Yazan L., Yap L. H., Wan Nor Hafiza W. A. G., How C. W., Abdullah R. (2015). Thymoquinone-loaded nanostructured lipid carrier exhibited cytotoxicity towards breast cancer cell lines (MDA-MB-231 and MCF-7) and cervical cancer cell lines (HeLa and SiHa). *BioMed Research International*.

[158] Abdelwahab S. I., Sheikh B. Y., Taha M. M. E. (2013). Thymoquinone-loaded nanostructured lipid carriers: preparation, gastroprotection, in vitro toxicity, and pharmacokinetic properties after extravascular administration. *International Journal of Nanomedicine*.

[159] Silachev D. N., Plotnikov E. Y., Zorova L. D. (2015). Neuroprotective effects of mitochondria-targeted plastoquinone and thymoquinone in a rat model of brain ischemia/reperfusion injury. *Molecules*.

